# Deubiquitinases in hematological malignancies

**DOI:** 10.1186/s40364-021-00320-w

**Published:** 2021-08-28

**Authors:** Hu Lei, Jiaqi Wang, Jiacheng Hu, Qian Zhu, Yingli Wu

**Affiliations:** Department of Pathophysiology, International Institute of Medicine, Shanghai Tongren Hospital/Faculty of Basic Medicine, Key Laboratory of Cell Differentiation and Apoptosis of the Chinese Ministry of Education, Shanghai Jiao Tong University School of Medicine, Shanghai, 200025 China

**Keywords:** Deubiquitinases, Hematological malignancies, Leukemia, Lymphoma, Multiple myeloma

## Abstract

Deubiquitinases (DUBs) are enzymes that control the stability, interactions or localization of most cellular proteins by removing their ubiquitin modification. In recent years, some DUBs, such as USP7, USP9X and USP10, have been identified as promising therapeutic targets in hematological malignancies. Importantly, some potent inhibitors targeting the oncogenic DUBs have been developed, showing promising inhibitory efficacy in preclinical models, and some have even undergone clinical trials. Different DUBs perform distinct function in diverse hematological malignancies, such as oncogenic, tumor suppressor or context-dependent effects. Therefore, exploring the biological roles of DUBs and their downstream effectors will provide new insights and therapeutic targets for the occurrence and development of hematological malignancies. We summarize the DUBs involved in different categories of hematological malignancies including leukemia, multiple myeloma and lymphoma. We also present the recent development of DUB inhibitors and their applications in hematological malignancies. Together, we demonstrate DUBs as potential therapeutic drug targets in hematological malignancies.

## Background

**Fig. 1:**
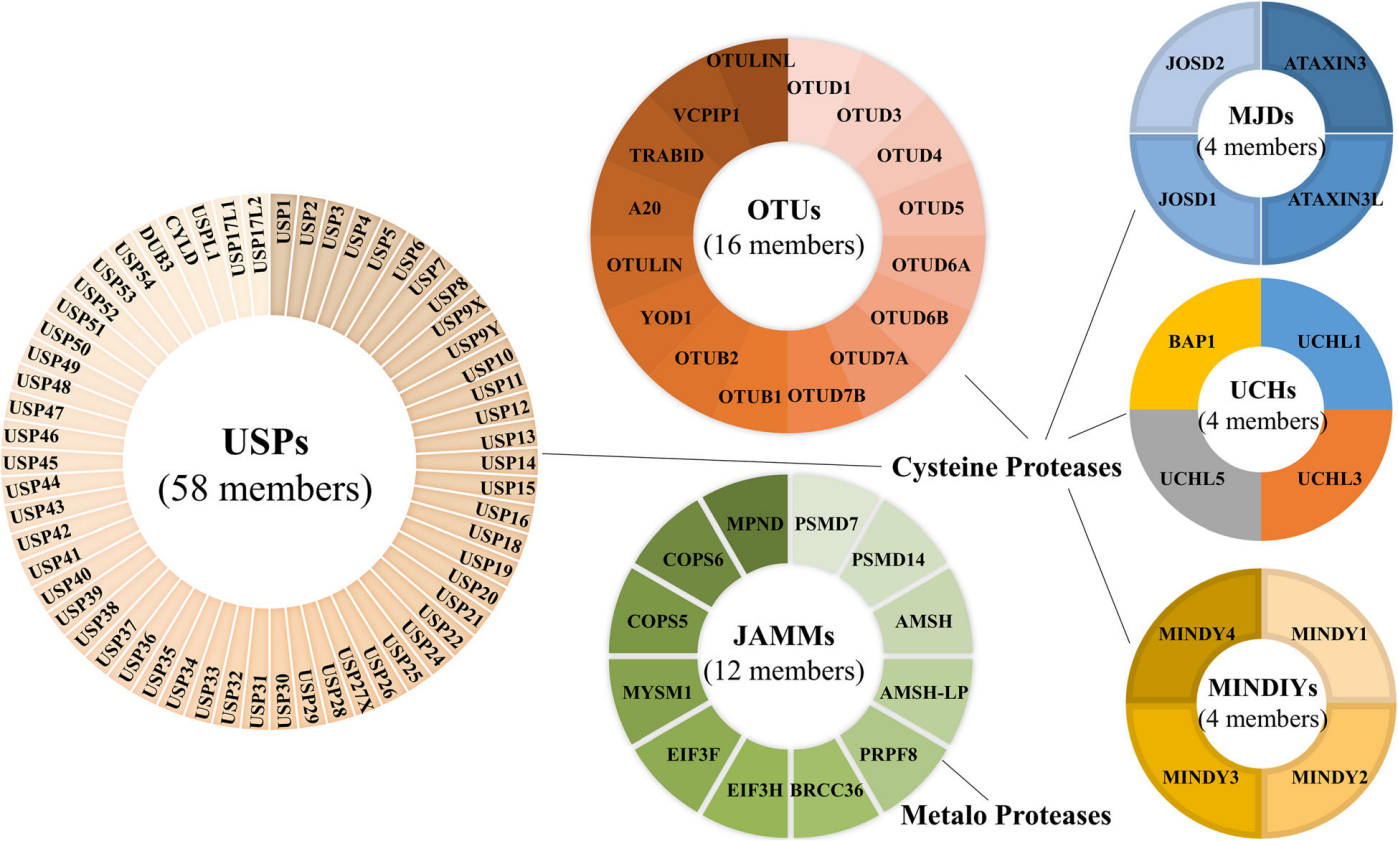
Human DUBs. Human DUBs are composed of cysteine proteases and metalo proteases. According to their domain structure, DUBs are classified in to six families: the ubiquitin-specific proteases (USPs), the ubiquitin carboxy-terminal hydrolases (UCHs), the ovarian tumor-related proteases (OTUs), the Machado-Joseph disease protein domain proteases (MJDs), the JAB1/PAB1/MPN-domain containing metallo-enzyme (JAMMs), and the motif interacting with Ub-containing novel DUB family (MINDYs)

The ubiquitin-proteasome system (UPS) regulates many cellular functions, including proteasomal degradation, selective autophagy, cell signaling, receptor trafficking and endocytosis, DNA damage response, cell cycle, cell survival, cell proliferation and cell death et al. [1–3]. The dysfunction or dysregulation of UPS is related to a series of pathologies and diseases, including hematological malignancies [4]. Inhibiting the activity of components of the UPS by bortezomib or carfilzomib has been shown as a therapeutic strategy for the treatment of multiple myeloma (MM) and mantle-cell lymphoma (MCL) [5]. Indeed, the essential components of the UPS consist of ubiquitinases, deubiquitinases and 26S proteasome [6]. Until now, many E3 ubiquitin ligases have been implicated in the pathogenesis of hematological malignancies [7–10]. Reversely, deubiquitinases (DUBs), contains ~ 100 family proteins, can remove the ubiquitin chains from the substrates and thereby control the stability, interactions or localization of the substrates [11, 12]. DUBs are classified in to six families according to their domain structure: the ubiquitin-specific proteases (USPs, 58 members), the ubiquitin carboxy-terminal hydrolases (UCHs, 4 members), the ovarian tumor-related proteases (OTUs, 16 members), the Machado-Joseph disease protein domain proteases (MJDs, 4 members), the JAB1/PAB1/MPN-domain containing metallo-enzyme (JAMMs, 12 members), and the motif interacting with Ub-containing novel DUB family (MINDYs, 4 members) (Fig. 1) [12]. Therefore, more and more evidences indicate that dysregulation of DUBs plays an important role in the pathogenesis of hematological malignancies [13, 14]. Also, some DUBs are important in the process of differentiation from hematopoietic stem cells (HSCs) to all blood-cell lineages, and the related disorders are associated with hematological malignancies [15–18]. However, most of DUBs and their roles in the progression of hematological malignancies have not been explored broadly. In this review, we will focus on the function of DUBs in different types of hematological malignancies and the development of targeted small molecule inhibitors.

### DUBs in chronic myeloid leukemia

**Fig. 2:**
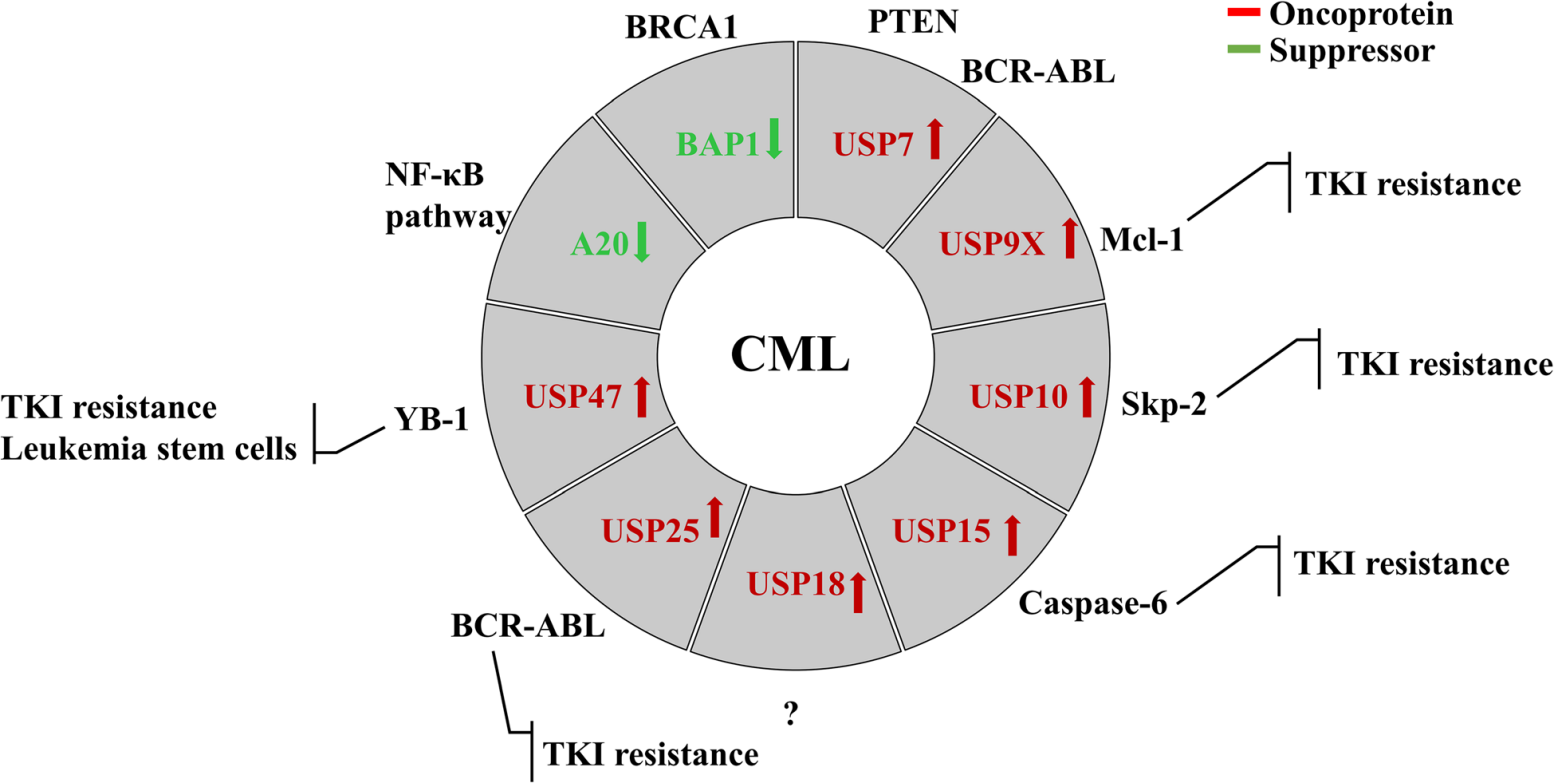
DUBs in chronic myeloid leukemia. The red arrows represent oncogenes and the green arrows represent tumor suppressors in CML. The surrounding text describes the substrates or signaling pathways or effects of DUBs

Chronic myeloid leukemia (CML) is a myeloproliferative neoplasm that accounts for approximately 15% of newly diagnosed cases of leukemia in adults [19]. BCR-ABL is responsible for 95% of all diagnosed CML cases, which acts as a constitutively active protein tyrosine kinase [20]. Delightfully, most of the CML patients can achieve complete remission after receiving tyrosine kinase inhibitors (TKIs) treatment, and some patients even discontinue the TKIs treatment to achieve treatment-free remission (TFR) [21, 22]. However, drug resistance and relapse restrict the application of TKIs in treating CML through BCR-ABL-dependent and -independent mechanism [23]. The BCR-ABL-dependent mechanism is mediated through *BCR-ABL* gene amplification, mutation of the ABL kinase domain and MDR1 upregulation [24]. The BCR-ABL-independent mechanism has still not been well understood until now. Leukemia stem cells (LSCs) in CML are not dependent on BCR-ABL kinase activity for their survival and are insensitive to TKIs in a BCR-ABL-independent manner, therefore leading to relapse [25]. In addition, the aberrant activation of RAS/MAPK or PI3K signaling pathways also contributes to BCR-ABL-independent TKI resistance [26, 27]. Some DUBs have been identified to be associated with BCR-ABL signaling pathway and others could overcome BCR-ABL-dependent or -independent TKI resistance. The DUBs related to CML are shown in Fig. 2.

USP7 is involved in DNA damage response, DNA replication, epigenetics and viral infections [28]. More importantly, USP7 has been identified as a potential therapeutic target for a variety of cancers due to its role in tumorigenesis [29]. Several small molecule specific inhibitors of USP7 have been developed [30, 31]. In CML, USP7 physically interacts with BCR-ABL and is phosphorylated (Tyr243) by BCR-ABL. Phosphorylated USP7 gains increased deubiquitinating activity, thus promoting the nuclear exclusion of phosphatase and tensin homolog (PTEN), which disrupts PTEN’s tumor suppressive function in CML cells [32, 33]. It is also demonstrated that USP7 interacts with BCR-ABL and blocks its polyubiquitination and degradation, thereby inhibiting its downstream signaling transduction [34]. Furthermore, USP7 is expressed mostly in the nucleus of normal CD34^+^ cells, but in primary CML CD34^+^ cells, it is expressed both in the nuclear bodies and cytoplasm [35]. Together, USP7 binds to BCR-ABL in the cytosol and regulates PTEN de-ubiquitination via a PML network in the nuclear bodies.

USP9X acts as an oncogene or tumor suppressor in human cancers, and it also plays a role in the context of normal development and the biological consequences of the disease [36]. USP9X inhibition by the small molecule WP1130 induces BCR-ABL ubiquitination and trafficking, leading to apoptosis in both imatinib-sensitive and -resistant CML cells. Although WP1130 inhibits USP9X activity, USP9X isn’t involved in the increased ubiquitination of BCR-ABL. USP9X silencing in CML cells decreases anti-apoptotic protein MCL-1, which increases sensitivity to imatinib and other apoptotic stimuli [37, 38].

USP10 is a deubiquitinase of S-phase kinase-associated protein 2 (SKP2), which interacts with BCR-ABL and is required for the activation of BCR-ABL in CML. SKP2, an E3 ligase, enhances the activity of BCR-ABL by promoting the K63-linked ubiquitination of BCR-ABL [39]. Although USP10 deubiquitinates and stabilizes SKP2, it cannot directly reduce the ubiquitination of BCR-ABL. Together, USP10 inhibition suppresses the proliferation of imatinib-sensitive and -resistant CML cells.

USP15 is involved in various cellular pathways associated with cancer and other related diseases [40]. USP15 is significantly down-regulated in CML, and its inhibition decreases de-ubiquitination of caspase-6 and promotes the degradation of caspase-6, which attenuates CML cell apoptosis and contributes to imatinib resistance [41].

USP18 (UBP43) is an ISG15-specific isopeptidase, the expression of which is activated by interferon (IFN) [42]. *Usp18*-deficient bone marrow cells show significant delay of CML development in BCR-ABL retroviral transduction/transplantation assay compared with wild-type ones. However, *Usp18* and *IFN receptor R1* (*Ifnar1*) double deficient bone marrow cells with p210 BCR-ABL transduction reverse the original resistance to CML disease development, which indicates the important role of type 1 IFN signaling in the resistance to CML development in *Usp18*-deficient bone marrow cells [43].

USP25 plays an important role in the regulation of innate immune response, autoimmunity and tumorigenesis by interacting with different tumor necrosis factor (TNF) receptor-related factor (TRAF) proteins [44]. BCR-ABL degradation has been considered as a strategy to overcome TKIs resistance, so inhibiting the deubiquitinases of BCR-ABL may be effective. USP25 deubiquitinates and suppresses the degradation of BCR-ABL, and its depletion inhibits BCR-ABL-mediated signaling and cell proliferation [45].

USP47 is involved in cell survival [46], cell proliferation [47, 48], DNA damage repair [23, 49, 50], epithelial-mesenchymal transition [51], and inflammation [52, 53]. It is demonstrated that *Usp47* knockout significantly prolongs the survival of BCR-ABL and BCR-ABL^T315I^-induced CML mice by reducing leukemia stem/progenitor cells. Mechanism studies have shown that USP47 deubiquitinates and stabilizes Y-box binding protein 1(YB-1) and participates in DNA damage repair in CML cells [23]. It is indicated that USP47 is required for BCR-ABL-induced CML and can overcome BCR-ABL-independent drug resistance.

A20 expression is significantly downregulated in CML CD34^+^ cells compared to normal bone marrow CD34^+^ cells. Overexpression of A20 inhibits cell proliferation, cell cycle, and promotes apoptosis in CML CD34^+^ cells. Furthermore, A20 overexpression significantly reduces the NF-κB signal pathways (P65 and IκBα phosphorylation) in K562 and CML CD34^+^ cells [54].

BAP1, down-regulated in CML at the transcriptional level, is a deubiquitinase interacting with the DNA repair regulator BRCA1 [55]. It is demonstrated that BAP1 is a major link with the BCR-ABL-induced downregulation of BRCA1 in CML.

Taken together, targeting some deubiquitinases, such as USP7, USP9X, USP10, USP15, USP25 and USP47, can effectively overcome TKIs resistance in CML, and some deubiquitinases play an important role in maintaining the self-renewal ability of CML stem/progenitor cells. For patients with TKIs intolerance or relapse, the use of deubiquitinase inhibitors alone or in combination with TKIs may be effective in solving clinical problems.

### DUBs in acute myeloid leukemia

**Fig. 3:**
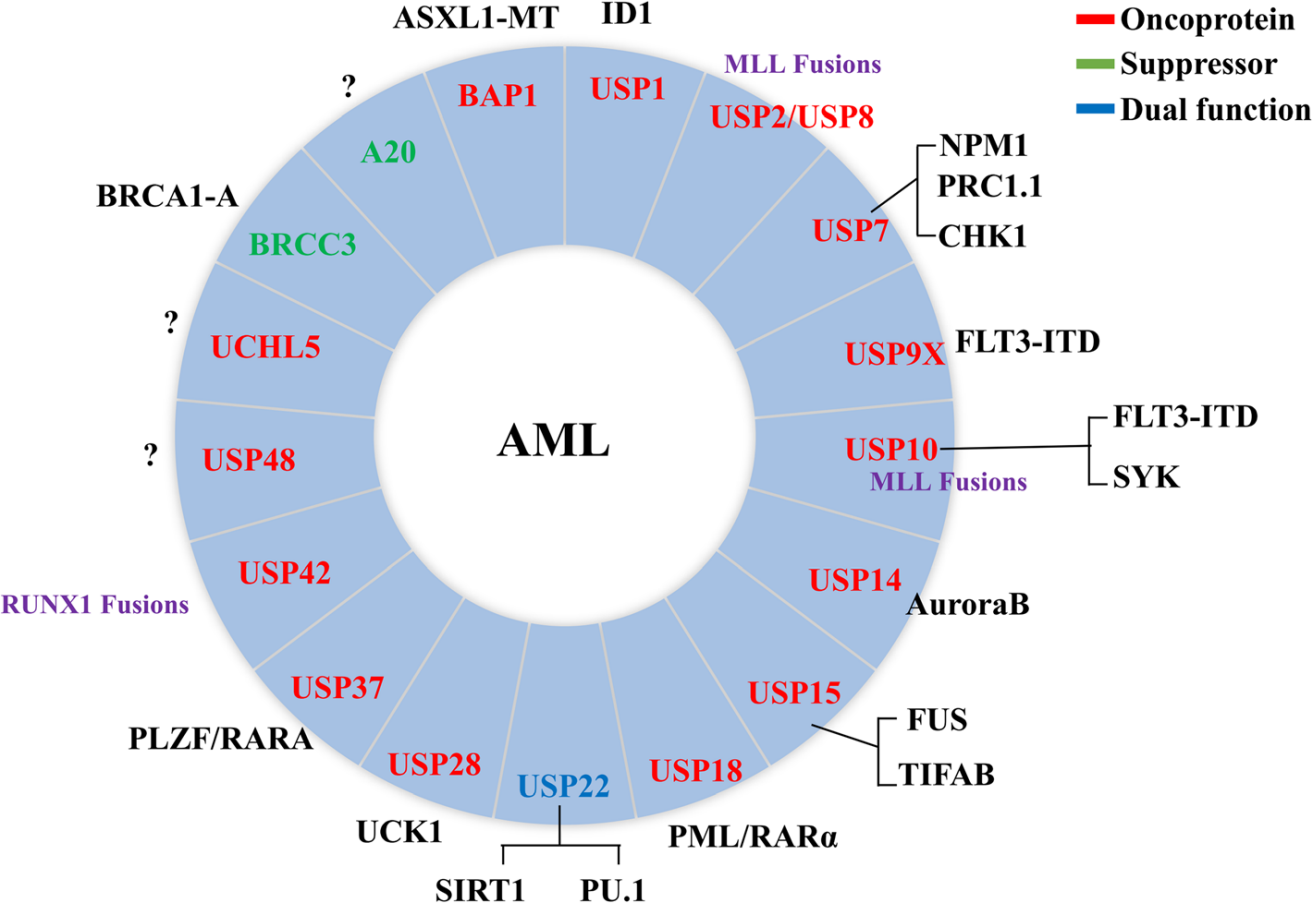
DUBs in acute myeloid leukemia. The red arrows represent oncogenes and the green arrows represent tumor suppressors in AML. USP22 acts as an oncogene or tumor suppressor in AML, depending on the context. The surrounding text describes the substrates or related proteins of DUBs in AML

Acute myeloid leukemia (AML) is a disease characterized by the clonal proliferation of primitive hematopoietic stem or progenitor cells. The abnormal differentiation of bone marrow cells leads to an increase in the number of immature malignant cells and a decrease in the number of differentiated red blood cells, platelets and white blood cells [56]. Although some new drugs are applied in the treatment of AML patients, the standard therapy for AML patients is still chemotherapy. However, some patients are intolerable for such treatment, especially elderly patients, while the effect seems limited [57–59]. Indeed, most patients receiving chemotherapy experience relapse, which is caused by chemo-resistant leukemia cells (RLCs) regrowth [60]. In addition, leukemia stem cells (LSCs) and drug resistance (FLT3 inhibitors, BCL2 inhibitors etc.) are still difficult problems for the treatment of AML [61–63]. Further research on the molecular mechanism of pathogenesis will contribute to the therapeutic effect in AML. The DUBs related to AML are shown in Fig. 3.

USP1 is frequently overexpressed in several cancer tissue types [64]. In AML cells, USP1 deubiquitinates inhibitor of DNA binding 1 (ID1) and rescues it from proteasome degradation, which plays a role in cellular transformation. USP1 inhibitor, SJB2–043, promotes ID1 degradation and inhibits primary AML cell growth by disrupting the homologous recombination [65].

USP2 is identified as a fusion partner of MLL (also known as KMT2A) in infant acute myeloid leukemia by using a whole transcriptome sequencing analysis [66]. Another study shows that acute leukemia patients are recurrently associated with MLL-USP2 fusion alleles as well as MLL fusion partnerships with USP8, AF4, and AF9 [67, 68]. MLL-USP2 fusion protein can potentially contribute to MLL-r leukemogenesis through three mechanisms: (a) USP2 stabilizes MLL to protect it from ubiquitin-proteasome degradation, (b) dominant negative inhibition of wild-type MLL transcription, and (c) regulate cell cycle, cell proliferation or other cell function by USP2.

USP7, which is known as a PTEN deubiquitinating enzyme, directly interacts with nucleophosmin (NPM1) /mutated nucleophosmin (NPMc+) in AML. NPM1 prevents USP7-mediated deubiquitination of PTEN in the nucleus, which promotes the shuttle of PTEN to the cytoplasm. PTEN is kept in the cytoplasm by USP7 deubiquitination activity, which is regulated by NPMc+ [69]. Furthermore, USP7 inhibition sensitizes AML to chemotherapy by interacting with and modulating CHK1 protein [70]. Also, USP7 deubiquitinases the non-canonical PRC1.1 polycomb complex to target loci and function in gene regulation in AML cells [71]. Together, USP7 is a promising therapeutic target in AML.

USP9X inhibits the K63-linked poly-ubiquitination of FLT3-ITD, while FLT3-ITD reversely phosphorylates USP9X to enhance ubiquitination and proteasomal degradation. Inhibition of USP9X by its inhibitor WP1130 or G9 shows potent anti-leukemia effects in FLT3-ITD-driven cells by blocking downstream signaling events of FLT3 [72].

USP10 is identified as a critical DUB to deubiquinate and stabilize FLT3-ITD more than wild-type FLT3 in AML. USP10 inhibition by HBX19818 shows anti-leukemia effect in FLT3-ITD positive AML cells and mouse models. It works synergistically with FLT3 inhibitors and overcomes FLT3 inhibitor resistance [73]. Another USP10 inhibitor, Wu-5, also shows anti-AML effect and overcomes FLT3 inhibitor resistance and synergistically enhances the anti-leukemia effect of crenolanib through targeting both FLT3 and AMPKα pathway [74]. Spleen tyrosine kinase (SYK), stabilized by USP10, is critical for AML transformation and maintenance of the leukemia clone in AML patients. Highly activated SYK is found in FLT3-ITD positive AML, which facilitates Myc transcriptional programs and is critical for TKI resistance [75, 76]. MLL-USP10 fusion has been identified in an adolescent case of relapsed AML-M5a with t (11,16) (q23;q24) [77]. Until now, USP2, USP8, and USP10 have been found to be fused with MLL, which indicates that DUBs may be potential targets of MLL-r leukemia.

The inhibitor of USP14 and UCHL5, b-AP15, inhibits organ infiltration in an AML mouse model [78]. Another compound NiPT, which potently inhibits USP14 and UCHL5 activity, induces cytotoxicity and proteasome inhibition in AML cells [79]. Aurora kinase B (Aurora B), a mitotic checkpoint kinase, has been found to be overexpressed in several types of leukemia [80]. USP14 inhibits chemotherapeutic drugs-induced apoptosis in leukemia cells by deubiquitinating Aurora B [81].

USP15 is highly expressed in human leukemia cells, which interacts with and stabilizes FUS in AML cells [15]. FUS, an RNA/DNA-binding protein, has been reported to promote HSC self-renewal [82]. During normal hematopoiesis, USP15 depletion impairs hematopoietic stem and progenitor cells (HSPCs) proliferation in vitro and reconstitution potential in vivo [15]. In MLL-AF9 leukemia, TIFAB deubiquitination regulated by USP15, decreases p53 signaling and correspondingly promotes leukemia cell function and development of leukemia [83].

USP18 was cloned from leukemia fusion protein AML1-ETO-expressing mice, which blocks cytokine-induced terminal differentiation of monocytic cells [84]. It is demonstrated that USP18 stabilizes PML/RARα protein and inhibits cell apoptosis in all-trans-retinoic acid (RA)-sensitive and RA-resistant acute promyelocytic leukemia (APL) cells [85]. However, other researchers found that USP18 could oppose UBE1L-dependent PML/RARα degradation by targeting the PML domain, rather than RA-dependent PML/RARα degradation [86].

USP22 mRNA expression is significantly induced in FLT3-ITD compared to FLT3-WT AML CD34^+^ cells by c-Myc, which leads to reduced ubiquitination and enhanced stability of SIRT1 [87]. In contrast, USP22 deficiency blocks myeloid differentiation, therefore promoting AML in Ras-driven myeloproliferative neoplasm. Mechanistically, USP22 deubiquitinates PU.1 and promotes its target genes expression [88]. Together, USP22 shows different functions in the process of leukemia transformation or pathology.

USP28 overexpression not only inhibits AML cell proliferation but also sensitizes AML cells to 5′-azacytidine (5′-AZA)-induced apoptosis. USP28 binds to uridine-cytidine kinase 1 (UCK1) via KLHL2 (an ubiquitin E3 ligase) and antagonizes KLHL2-mediated poly-ubiquitination of UCK1, which has an established role in activating 5′-AZA [89].

USP37 interacts with the promyelocytic leukemia zinc finger (PLZF)/the retinoic acid receptor alpha (RARA) fusion proteins and stabilizes its protein levels. Acute promyelocytic leukemia (APL) patients with PLZF/RARA fusion protein are largely resistant to all-trans retinoic acid (ATRA) treatment and with poor prognosis [90].

USP42 (intro 1) was found to be fused with RUNX1 (intron 7) (t (7,21)(p22;q22)/RUNX1-USP42) in AML, but the function of USP42 was unknown [91]. However, later studies showed that exon 6 or exon 7 of RUNX1 was fused to exon 3 of USP42 with aberrant expression of CD56 and CD7 [92, 93]. AML patients with RUNX1-USP42 show poor prognosis [94]. It is suggested that USP42 may play an important role in AML-ETO positive AML patients.

USP48 is involved in ATRA-induced APL cell differentiation. ATRA treatment in APL cell lines causes nucleus translocation in 24 h and deregulation of USP48. USP48 overexpression inhibits APL cells proliferation and promotes ATRA-mediated differentiation [95].

BRCA1/BRCA2-containing complex 3 (BRCC3) is a member of the JAMMs family capable of cleaving Lys-63 linked poly-ubiquitin chain [96]. BRCC3 mutations result in improved proliferation in AML1-ETO positive AML cell lines and unlimited self-renewal in mouse hematopoietic progenitor cells in vitro. However, BRCC3-mutated AML1-ETO positive AML patients show favorable outcome, since BRCC3 inactivation may lead to an impaired capability of the BRCA1-A complex to repair DNA damage and subsequently higher sensitivity to DNA damaging chemotherapy [97]. There may be significant differences in the pathogenesis of AML between mice and humans.

A20 expression was up-regulated in AML derived DCs (AML-DCs), which are differentiated from AML leukemia cells [98]. Also, A20 expression was increased during monocyte-macrophage differentiation (THP-1 cell) [99]. However, the expression of A20 was lower in T cells from patients with AML than those in the healthy controls [100]. Together, A20 acts as a tumor suppressor in AML.

BAP1 depletion inhibits the growth of myeloid leukemia cells with ASXL1 mutations or MLL-fusions. ASXL1 mutations or MLL-fusions are associated with poor prognosis in a variety of myeloid neoplasms. Mechanistically, the C-terminally truncated mutant ASXL1 (ASXL1-MT) forms a complex with BAP1 and induces the up-regulation of HOXA gene and IRF8 by removing H2AK119 ubiquitination [101, 102].

Taken together, some deubiquitinases, such as USP9X, USP10, USP18 and USP37, are directly associated with leukemic fusion proteins. Also, other deubiquitinases, such as USP2, USP8, USP10 and USP42, fuse with leukemia-associated genes, indicating that deubiquitinases play an important role in the pathogenesis of AML.

### DUBs in acute lymphoblastic leukemia

**Fig. 4:**
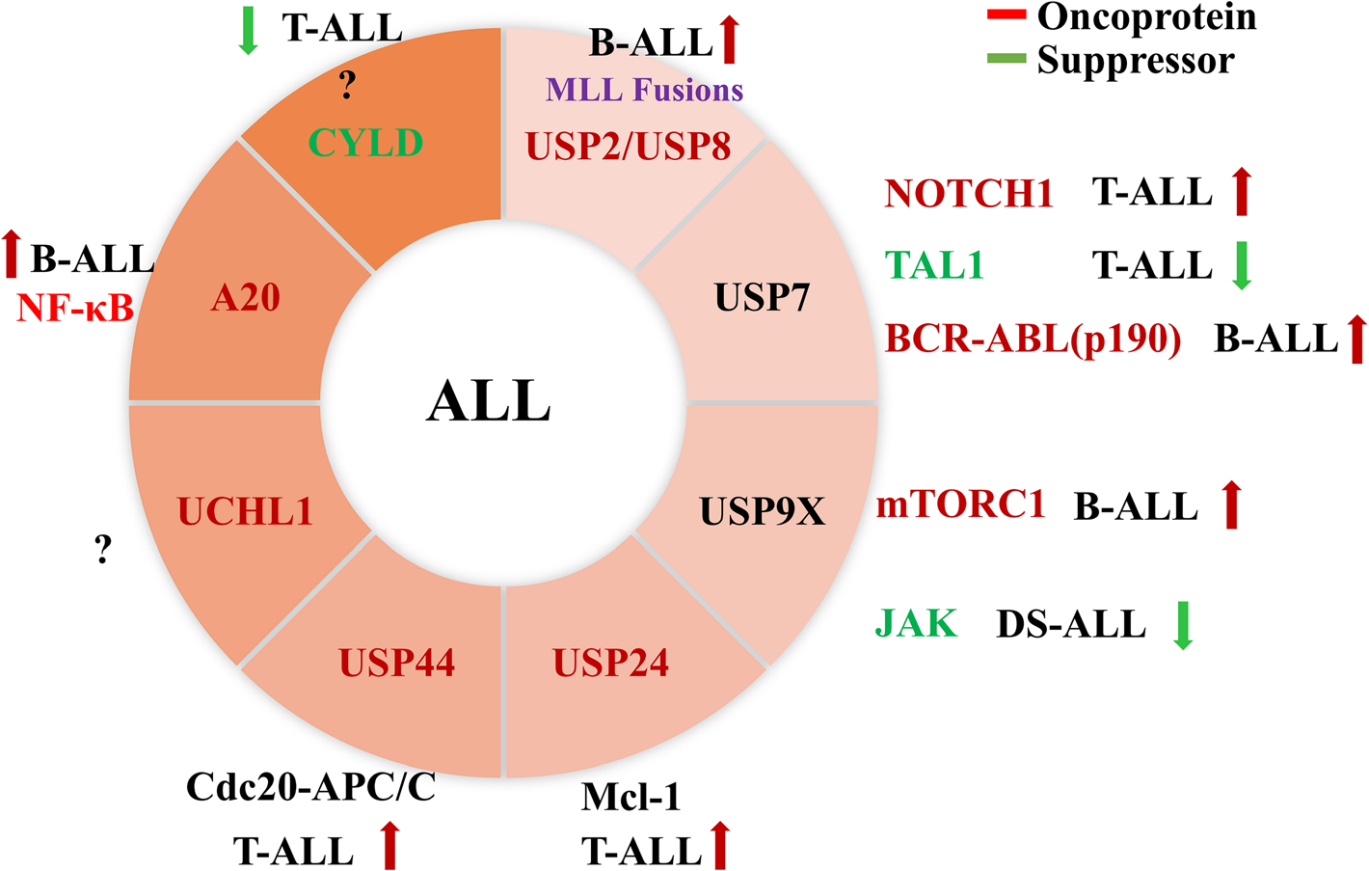
DUBs in acute lymphoblastic leukemia. The red arrows represent oncogenes and the green arrows represent tumor suppressors in ALL. The surrounding text describes the substrates or signaling pathways involved in DUBs of different types of ALL

Acute lymphoblastic leukemia (ALL) is caused by malignant proliferation of lymphoid cells (including T and B cells) blocked at an early stage of differentiation that can invade bone marrow, blood and extramedullary sites [103]. The first-line treatment for ALL is chemotherapy and typically includes three phases: induction, consolidation, and maintenance [104]. Radiation therapy can be used for patients with evidence of central nervous system (CNS) or testicular leukemia, while allogeneic hematopoietic cell transplantation and growth factor therapy are complementary to all treatments [105, 106]. New immunotherapeutic strategies, such as monoclonal antibodies and chimeric antigen receptor (CAR) T cells, are also being developed [107, 108]. However, the management of ALL in adults and elderly ALL patients (> 60 years old) is still challenging despite the great progress made in the past decades. The DUBs related to ALL are shown in Fig. 4.

USP2/USP8-MLL rearrangement is identified in infant patients with B-ALL [66, 109]. The majority of infant ALL is characterized by MLL rearrangements (~ 70 to 80%, with a poor prognosis) in acute lymphoblastic leukemia. Targeting USP2/USP8 may be important to improve the outcomes of MLL-rearranged leukemia.

USP7 is identified as a susceptible locus for T-ALL in a genome-wide association study (GWAS) [110] and is significantly up-regulated in T-ALL cells [111]. Mechanistically, USP7 deubiquitinates and stabilizes NOTCH1, which leads to the decrease of the transcriptional levels of NOTCH1 targets and blocks T-ALL cell growth [111, 112]. However, USP7 is frequently mutated in pediatric T-ALL, with somatic heterozygous loss-of-function mutations (haploinsufficiency) predominantly affecting the subgroup that has aberrant TAL1 oncogene activation. Haploinsufficiency of USP7 promotes cell growth and transcriptionally down-regulates E-proteins targets by interacting with TAL1 [113]. Specifically, USP7 interacts with p190 BCR-ABL in Philadelphia chromosome-positive (Ph+) ALL, and decreased USP7 activity is associated with p53 protein stability [114].

USP9X inhibition reduces leukemia cell growth via repressing mTORC1 activity, enhances spontaneous apoptosis and overcomes glucocorticoid resistance in B-ALL [115]. However, USP9X level is positively associated with survival in T-ALL patients and knockdown of USP9X does not induce apoptosis and growth inhibition in T-ALL cells [116]. It is reported that pharmacological or genetic inhibition of USP9X, as well as treatment with low-dose ruxolitinib, may promote the survival of CRLF2-positive B-ALL with down syndrome (DS-ALL) cells, potentially by restricting JAK signaling [117]. These findings suggest that USP9X may play different roles in different types of ALL.

USP24 inhibition significantly induces growth inhibition and apoptosis in T-ALL cells. WP1130 blocks USP24 activity by directly interacting with the activity site pocket of USP24 in T-ALL cells. Mechanistically, USP24 inhibition regulates Mcl-1 stability to accelerate the collapse of mitochondrial transmembrane potential [116].

USP44 expression is elevated in T-ALL. USP44 overexpression leads to whole chromosomal instability, as well as increased cyclin B prior to mitosis, via regulating Cdc20-APC/C activity [118].

UCHL1 is decreased in the differentiation process of the lymphoblastic leukemia cell line, and its expression is independent of the apoptotic pathway [119]. However, the mechanism by which UCHL1 is involved in differentiation has not been elucidated.

A20 is expressed at high levels in ALL patients and cell lines, which promotes proliferation, regulates cell cycle progression and induces chemotherapy resistance [120]. A20 is also overexpressed in adult B-ALL, which may be related to the pathogenesis of B-ALL [121].

CYLD is significantly down-regulated in primary T-ALL [122]. Overexpression of CYLD could abolish the ability of constitutively active Notch1 in T-ALL [123]. Interestingly, cleaved CYLD overexpression in normal human peripheral blood mononuclear cells has the inherent capacity to program the genome of these cells resulting in T-cell lineage ALL [124]. Together, CYLD acts as a tumor suppressor in ALL.

Taken together, USP7, USP9X, USP24 and USP44 have been shown to promote the development of ALL as oncogenic genes. However, USP7 and USP9X also act as tumor suppressor genes in ALL. Therefore, targeting different deubiquitinases may have a role in certain types of ALL.

### DUBs in chronic lymphocytic leukemia

Chronic lymphocytic leukemia (CLL) is the most frequent adult leukemia, which is initiated by specific genomic alterations that impair apoptosis of clonal B-cells [125]. With the development of kinase inhibitors that target B-cell receptor signaling, CLL therapy has transformed during the past decade. For example, Bruton’s tyrosine kinase (BTK) and isoform-selective phosphatidylinositol 3-kinase (PI3K) inhibitors disrupt B-cell receptor signaling. Besides, BCL2 (B-cell lymphoma 2) has emerged as another important therapeutic target [126]. New molecularly targeted drugs have shown good advantages over the previous chemotherapy regimens or monoclonal antibody regimens for elderly and high-risk CLL patients. However, drug resistance, side effects and high costs are currently existing problems.

USP7 is overexpressed in CLL compared with normal donors. Inhibition of USP7 partially impairs DNA repair by homologous recombination, which disrupts the stability of E3 ligase RAD18 and leads to the accumulation of DNA damage, therefore killing CLL cells independently of ataxia telangiectasia mutated (ATM) and p53 [127, 128]. USP7 is also shown to promote PTEN delocalization from the nucleus with consequent loss of part of its tumor suppressive function in a p53 dispensable manner [129]. Together, targeting USP7 is an effective strategy to kill CLL cells.

CYLD is considerably down-regulated in CLL cells compared to normal B cells, which correlates with lower overall survival (OS) in CLL patients [130, 131]. Lymphoid enhancer-binding factor 1(LEF1), a downstream effector of the Wnt/β-catenin pathway, can repress the transcription of CYLD and down-regulated CYLD inhibits TNF-α-induced necroptosis [130, 132]. Moreover, alternative splicing of CYLD is detected in many B-cell CLL patients’ samples, which can lead to CD5^+^ B-cell expansion through sustained NF-κB signaling [133].

A20 negatively regulates NF-κB activity in several types of B-cell malignancies. However, neither mutations nor aberrant DNA methylation is found at the A20 locus during gene analysis in CLL cells, while the expression of A20 is normal [134, 135]. These results suggest that A20 may not play a significant role in the pathogenesis of CLL.

### DUBs in multiple myeloma

**Fig. 5:**
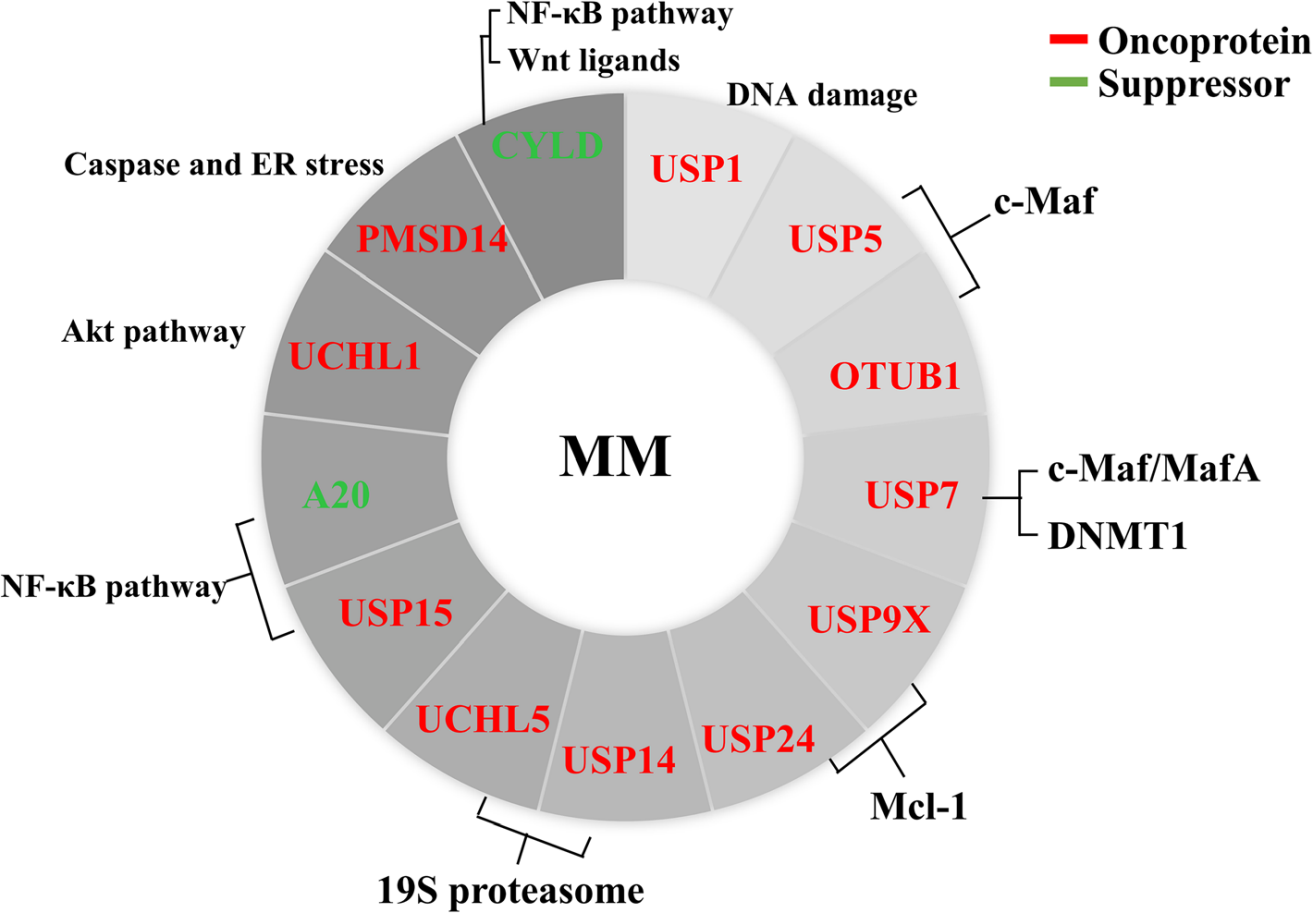
DUBs in multiple myeloma. The red arrows represent oncogenes and the green arrows represent tumor suppressors in MM. The surrounding text describes the substrates or signaling pathways of DUBs in MM

Multiple myeloma (MM) is the second most common hematological malignancy characterized by the accumulation of monoclonal plasma cells that produce M protein in the bone marrow [136]. The use of novel agents (such as proteasome inhibitors, immunomodulatory drugs and antibodies targeting cell surface molecules), as well as high-dose therapy and autologous stem cell transplantation (ASCT) in younger patients, has significantly improved the prognosis of patients with multiple myeloma [137]. However, most patients experience multiple relapses and eventually die of the disease itself or treatment-related complications, especially the elderly patients [138]. The discovery of new pathogenesis will help to develop new therapeutic drugs to overcome the problems of drug resistance and recurrence. Reports on the mechanisms by which deubiquitinating enzymes regulate the pathogenesis of MM have revealed the great potential for targeting deubiquitinates. The DUBs related to MM are shown in Fig. 5.

USP1, which participates in DNA damage response and cell differentiation, is overexpressed in some MM cases and is associated with poor prognosis. SJB3-019A is a selective inhibitor of USP1, which can decrease cell viability, trigger apoptosis, overcome bortezomib resistance in MM cells, and down-regulate renewal/survival related proteins and even promote MM stem cells differentiation [139].

USP5 and OTUB1 stabilize c-Maf and promote myeloma cell proliferation and survival [140–143], which indicates that USP5/c-Maf or OTUB1/c-Maf axis could be a potential target for myeloma therapy. Mebendazole down-regulates the expression of USP5 and disrupts the interaction between USP5 and c-Maf, which results in an increase in the level of c-Maf ubiquitination and subsequent c-Maf degradation [140]. MiR-125a inhibits the expression of USP5, thus reducing the proliferation and survival of MM cells [144]. Bortezomib-induced peripheral neuropathy (BIPN) is one of the most serious side effects associated with bortezomib treatment [145]. Interestingly, USP5 is found to be upregulated during this pathological process to stabilize Cav3.2, a T-type calcium channel essential for BIPN, in the mouse model [146]. Nanchangmycin (Nam), a polyketide antibiotic, is identified to inhibit c-Maf activity in the presence of OTUB1, indicating that Nam has the potential to treat MM by targeting the OTUB1/c-Maf axis [147].

USP7 overexpression predicts poor prognosis and USP7 inhibition overcomes bortezomib resistance in MM [148, 149]. USP7 is involved in bortezomib resistance by stabilizing NEK2 or repressing IκBα, and thus activating NF-κB signaling pathway [149, 150]. USP7 also promotes MM cell growth and inhibits cell apoptosis through other mechanisms. For example, USP7 stabilizes transcription factor Maf family members (c-Maf and MafA), which are highly expressed in MM and contribute to the invasion, adhesion and migration of MM cells [151]. USP7 stimulates DNA methyltransferase 1 (DNMT1) activity and blockade of USP7 enhances anti-MM activity of oxidative epigenetic agent RRx-001 [152]. Similarly, P5091, another USP7 inhibitor, inhibits MM cell growth and increases cell apoptosis both in vitro and in vivo [148, 151]. Interestingly, other USP7 inhibitors upregulate the transcription of genes that are normally silenced by the epigenetic suppressor complex polycomb inhibitor 2 (PRC2) and enhance the activity of PIM and PI3K inhibitors as well as DNA-damaging agents [153].

USP9X can remove the Lys48-linked poly-ubiquitin chains on Mcl-1 and thus prevent its degradation. It also promotes cell survival and its overexpression in MM is associated with poor clinical outcomes [154, 155]. USP24 can also stabilize Mcl-1 and promote MM cell survival independent of USP9X. WP1130, a small molecule inhibitor targeting USP9X, induces MM cell apoptosis both in vitro and in vivo [156]. Other leading compounds T5165804 and CP2005 inhibit USP9X activity at higher nanomolar potency against MM cell lines, compared with WP1130 [157].

USP14 and UCHL5, targeting the 19S regulatory subunit of the proteasome and affecting the proteasomal uptake of protein substrate for degradation, are highly expressed in MM and their inhibition by b-AP15 overcomes bortezomib resistance [78, 158, 159]. USP14 is involved in cell adhesion-mediated drug resistance (CAM-DR) of multiple myeloma cells by acting as a bridge between Bcl-xl apoptotic pathway and Wnt-signaling pathway [160].

USP15 expression is upregulated in MM patients compared with normal health ones. USP15 silencing induces MM cell proliferation inhibition and apoptosis via inhibiting NF-κB pathway. In turn, NF-κB p65 can thus promote USP15 expression, which forms a positive feedback loop [161].

UCHL1 is highly expressed in many MM cases and acts as a poor prognostic factor [162, 163]. UCHL1 impairs mTORC1 activity and increases mTORC2-mediated phosphorylation of the proliferative kinase Akt, and thus promotes the survival of MM cell [164].

PMSD14/Rpn11 is a component of the 26S proteasome, a multi-protein complex that catalyzes the degradation of ubiquitinated intracellular proteins [165]. Inhibition of PMSD14 activates the caspase cascade and endoplasmic stress response signals to trigger MM cell apoptosis and overcome bortezomib resistance [166].

Aberrant activation of the NF-κB pathway has been observed in MM [167], while CYLD is a negative regulator of NF-κB pathway, and the status of CYLD at 16q12 is highly correlated with the clinical outcome of MM patients [168]. In addition to affecting NF-κB signaling, CYLD loss or low expression also sensitizes MM cells to Wnt ligands, indicating another possible tumor-suppressive mechanism of CYLD in MM [169]. Moreover, it’s worth noting that the inhibitory effects of proteasome inhibitors on NF-κB signaling, which could be observed during MM treatment, might be mediated, at least in part, by the cellular accumulation of CYLD [170].

A20, a suppressor of the NF-κB pathway, is frequently down-regulated in MM, as a result of gene copy number reduction [171]. Berbamine treatment results in an increased expression of A20, which inhibits the proliferation of MM cells [172].

Taken together, deubiquitinase inhibitors have been shown to be effective in overcoming bortezomib resistance, so the combination of deubiquitinase inhibitors with existing therapies will become a new therapeutic strategy.

### DUBs in Hodgkin lymphoma

Hodgkin Lymphoma (HL) is a type of lymphoid malignancy derived from B cells of the germinal center or postgerminal center, which is characterized by a low number of malignant cells and a high number of immune effector cells in the tumor microenvironment [173]. Primary HL can be cured with radiation therapy and multiagent chemotherapy, and even recurrent or refractory HL can be effectively treated or cured with high-dose chemotherapy and autologous hematopoietic stem cell transplantation. In addition, immunotherapy with antibody-drug conjugates and immune checkpoint inhibitors have been shown to be effective against HL [174]. Due to the satisfying therapeutic effects of current therapies against HL, there are few studies on deubiquitination enzyme in HL.

Classical Hodgkin lymphoma (cHL), a subtype of HL accounting for 95% cases, is characterized by the presence of less than 1% malignant mononucleated Hodgkin and multinucleated Reed-Sternberg cells (HRS cells) mixed with nonneoplastic cells [175]. NF-κB signaling pathway, which is regulated by A20 and CYLD, is very important for the survival and proliferation of HRS cells [176]. Mutation or deletion of A20 gene exists in nearly 40% of HL cases, contributing to constitutive NF-κB activity in cHL cells, while A20 reconstitution confers cytotoxicity to A20-deficient cHL cells [177–179]. Deletion or DNA copy number loss of CYLD are detected in one of four commonly used cHL cell lines and biallelic gene mutations of CYLD are detected at low frequency [180, 181], suggesting that impaired CYLD function may contribute to some cHL cases.

### DUBs in non-Hodgkin lymphoma

**Fig. 6:**
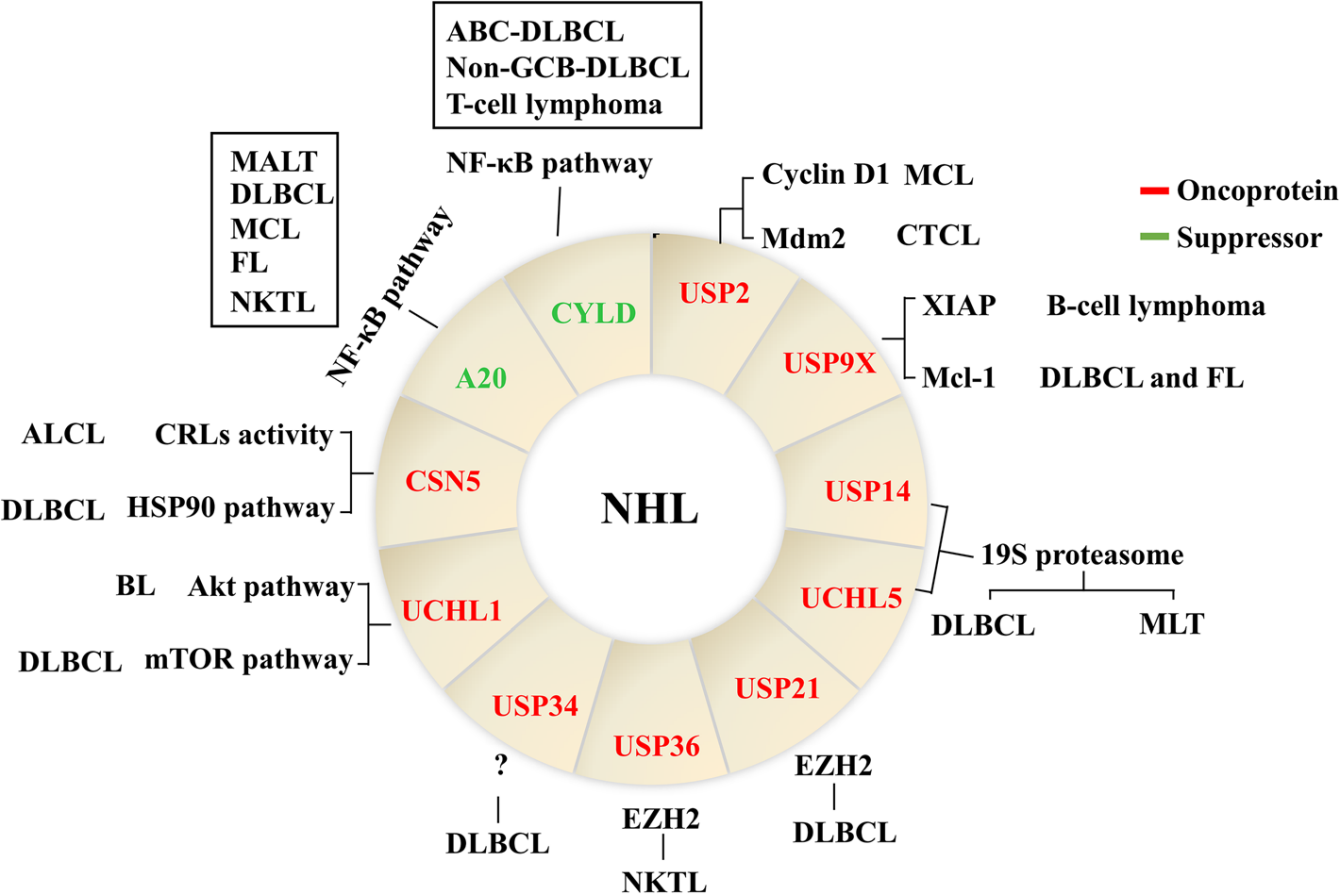
DUBs in non-hodgkin lymphoma. The red arrows represent oncogenes and the green arrows represent tumor suppressors in NHL. The surrounding text describes the substrates or signaling pathways involved in DUBs of different types of lymphoma. CRLs: cullin-RING E3 ubiquitin ligases

Non-Hodgkin Lymphoma (NHL) accounts for about 90% of all lymphomas, which can be subdivided into B-cell lymphomas and T-cell/ NK-cell lymphomas. The classification of NHL is complex and constantly evolving, with more than 80 different subtypes listed in the latest classification of the World Health Organization [182, 183]. Each type of NHL has its unique histological and biological characteristics, as well as different treatment strategies and clinical results, making NHL a complex disease [184]. From the perspective of treatment, NHL is divided into high grade and low grade lymphoma. Low grade NHL patients can be cured by surgical excision or radiotherapy, but most patients appear in advanced stages or transform to high grade disease [185]. For high grade NHL patients, chemotherapy is still the main treatment method, and some patients will also receive radiotherapy. For B cell-derived lymphomas, CD20 monoclonal antibody immunotherapy will also be used in combination [186, 187]. However, drug resistance and relapse during or after chemotherapy are major problems need to be solved, especially for T-cell/ NK-cell lymphomas. The DUBs related to NHL are shown in Fig. 6.

USP2 inhibition by ML364 induces an increase in cellular cyclin D1 degradation and causes cell cycle arrest in mantle cell lymphoma (MCL) cell line (Mino cell) [188]. In addition, USP2 stabilizes Mdm2, which antagonizes the pro-apoptotic activity of p53 and possibly contributes to therapeutic resistance in cutaneous T-cell lymphoma (CTCL) [189].

USP9X was found to be highly expressed in samples obtained from patients with aggressive B-cell lymphoma. Knockdown of USP9X leads to suppressed lymphoma growth and increased sensitivity to chemotherapy by destabilizing X-linked inhibitor of apoptosis protein (XIAP) in B-cell lymphoma, independently of Mcl-1 [190]. In diffuse large B cell lymphoma (DLBCL) and follicular lymphoma (FL), USP9X deubiquitinates Mcl-1 and protects it from degrading, while high-level Mcl-1 is associated with malignant B-cell proliferation and poor outcomes in B-cell lymphoma patients [154].

USP14 and UCHL5 are aberrantly expressed in the cytoplasm of DLBCL cells [191]. USP14 and UCHL5 inhibition by b-AP15 could induce cell apoptosis and suppress cell migration in both ABC- and GBC-subtypes of DLBCL [192]. In addition, USP14 inhibitor IU1 leads to tumor regression in malignant lymphomas of thymus (MLT) mouse model [193].

USP21 is overexpressed in DLBCL and promotes cell proliferation by maintaining the stability of enhancer of zeste homolog2 (EZH2), an oncogenic molecule in NHL [194, 195]. Targeted inhibition of USP21 activity resulted in a reduction in EZH2 and thus inhibited cell growth, independent of EZH2 mutations. Another DUB targeting EZH2, USP36, was found in nasal natural killer /T cell lymphoma (NKTL). USP36 stabilizes EZH2 by removing the K222-linked poly-ubiquitin chain from EZH2 [196].

USP34 expression is significantly higher in DLBCL than that in reactive lymphatic hyperplasia. USP34 overexpression is associated with age, germinal center B-cell-like (GCB) subtypes, multiple extranodal involvements, and higher International Prognostic Index (IPI) scores in DLBCL [197]. Also, USP34 gene amplification was detected in transformed DLBCL (tDLBCL) [198].

UCHL1 was detected to be highly expressed in both Burkitt’s lymphoma (BL) and DLBCL, which is correlated with a poor clinical outcome [199]. Knockdown of UCHL1 using specific shRNA in BL cells leads to reduced cell proliferation, as well as strong LFA-1-dependent homotypic adhesion [200]. Mechanistically, aberrantly expressed UCHL1 could activate the Akt pathway via down-regulating the antagonistic phosphatase PHLPP1 and Akt signaling [201], as well as promoting mTORC2 assembly [202]. At the same time, UCHL1 can bypass mTORC1 to promote the assembly of eIF4F, a translational regulator downstream to Akt and mTOR, which is required for MYC-driven B-cell malignancy in mice [202].

CSN5 is identified the protease as a positive regulator of oncogenes and negative regulator of tumor suppressors [203–205]. CSN5i-3, a CSN5 inhibitor, inhibits the growth of human-derived cell line SU-DHL-1 (anaplastic large-cell lymphoma, ALCL) in a xenograft model [206]. Also, CSN5 knockdown inhibits the growth of DLBCL cells partially through the CSN5-HSP90 pathway [207].

A20 mutations and/or deletions are linked with various subtypes of B-cell or T-cell lymphomas, including mucosal-associated lymphoid tissue (MALT) lymphoma, DLBCL, MCL, FL and NKTL [208–210]. In NKTL, monoallelic deletion of A20 occurs in 18% cases, while no biallelic deletion was detected [175]. A20 mutations occur in more than 50% of ABC-DLBCL and a small fraction of GCB-DLBCL [211]. Up-regulating A20 with phorbol myristate acetate (PMA) and lonomycin (IONO) could effectively induce cell cycle arrest at G0/G1 phase, suppress cell proliferation and induce apoptosis in OCI-LY1, a DLBCL cell line [212]. Re-expression of A20 in DLBCL cell lines carrying biallelic inactivation of the A20 gene leads to cell apoptosis and impairs NF-κB signaling [211]. After A20 knockdown, NF-κB signaling in T cells can be activated by CD3 alone, rather than CD3/CD28 co-stimulation [213]. However, A20 deficiency is not a prognostic factor in all of our NKTL cohorts. It is associated with shorter PFS in the high IPI subgroup [175].

The cleavage of CYLD by MALT1 is blocked by the selective inhibitor of MALT, which inhibits the growth of ABC-DLBCL cells both in vitro and in vivo [214]. CYLD anomalies are also detected in adult T-cell leukemia/lymphoma (ATLL) cells, as hyper-phosphorylated CYLD losses its function of removing the ubiquitin chains on RIP1, leading to continuous activation of the NF-κB pathway [215]. Furthermore, CYLD phosphorylation down-regulation by BTK inhibitors induces cell apoptosis and tumor growth inhibition in non-GCB-DLBCL, especially in the rituximab-resistant relapsed/refractory cases [216].

Taken together, there are many types of NHL, and some deubiquitinases have shown great significance in certain types of NHL. However, the pathogenesis role of deubiquitinases in NHL and relative combination therapy need further investigation.

### DUB inhibitors in hematological malignancies

Unlike E3 ligases, most of DUBs can be drugged according to their cysteine proteases or metalo proteases [12, 217]. To date, several DUBs, particularly those in the USP family, have been successfully targeted as small molecule inhibitors showing cytotoxic effects in vitro and in vivo. In addition to their potential therapeutic implications, the use of inhibitors has contributed greatly to understanding the role DUBs plays in various processes. In Table 1, we list the inhibitors of DUBs that have been reported so far and whether they are used in hematological malignancies.

**Table 1:** DUB inhibitors in hematological malignancies

Compound	Structure	DUBs target	Year reported and reference	Hematological malignancies and reference
PR-619		Many DUBs	2011 [218]	/
SJB3-019A		USP1	2013 [65]	AML [65], MM [139], B-ALL [219]
SJB2–043				AML [65]
ML323			2014 [220]	CML [221]
ML364		USP2, USP8	2016 [188]	Lymphoma [188]
Q29		USP2	2015 [222]	/
LCAHA			2017 [223]	/
STD1T			2018 [224]	/
6TG			2018 [225]	/
Vialinin A		USP4, USP5, UCHL1	2013 [226]	/
P5091		USP7, USP47	2012 [148, 227]	MM [148, 151], MDS [228]
P22077			2013 [229]	CML [23], MDS [228], AML [73]
HBX41108		USP7	2009 [230]	/
GNE-6776, GNE-6640			2017 [30]	AML [30]
XL188			2017 [231]	/
FT671, FT827			2017 [232]	/
P217564			2017 [233]	/
Compound 4			2017 [31]	/
XL177A			2020 [234]	/
HY50737A, HY50736		USP7, USP8	2010 [235]	/
HBX19818		USP7, USP10	2012 [236]	AML [76]
WP1130		USP5, USP9X, USP14, UCHL1, UCHL5	2007 [237]	CML [37, 38], MM [141, 156], Lymphoma [238], AML [239]
9-(Ethoxyimino)-9H-indeno[1,2-b]pyrazine-2,3-dicarbonitrile		USP8	2010 [235]	/
T5165804, CP2005		USP9X	2014 [157]	MM [157]
Wu-5		USP10	2020 [74]	AML [74]
Spautin-1		USP10, USP13	2011 [240]	CML [39, 241], AML [242]
EOAI3402143		USP5, USP9X, USP24	2015 [156]	MM [156]
Mitoxantrone		USP11	2013 [243]	AML [244], Lymphoma [245]
b-AP15		USP14, UCHL5	2011 [78]	AML [78, 81], MM [159, 246], DLBCL [192], MCL [247]
VLX1570			2015 [248]	MM [248, 249], ALL [250]
Auranofin			2014 [251]	MM [252, 253], ALL [254], CLL [255], CML [256], AML [257], Lymphoma [258, 259]
IU1		USP14	2010 [260]	/
IU1–248			2018 [261]	/
RA-9		Proteasome-Associated DUBs	2014 [262]	/
GSK2643943A		USP20	2016 [263]	/
NCI677397		USP24	2021 [264]	/
AZ1		USP25, USP28	2017 [265]	/
15-oxospiramilactone		USP30	2014 [266]	/
MF-094			2018 [267]	/
LDN57444		UCHL1, UCHL3	2003 [268]	/
LDN91946		UCHL1	2007 [269]	/
Z-VAE(Ome)-fmk			2012 [270]	/
TCID		UCHL3	2012 [271]	/
NSC112200		TRABID	2012 [272]	/
BC-1471		AMSH	2017 [273]	/
Capzimin		PSMD14/Rpn11	2017 [274]	MM [274]
Thiolutin			2017 [275]	/
O-phenanthroline			2017 [166]	MM [166]
SOP10			2018 [276]	/
CSN5i-3		CSN5	2016 [206]	Lymphoma [206]
iBAP		BAP1	2021 [102]	Leukemia [102]

Although DUBs are promising drug targets for the treatment of hematological malignancies, the development of DUB specific inhibitors has not been an easy task. This is because: 1) DUBs show complex structural characteristics in their catalytic domain and similarities among family members, which makes it difficult to target; 2) Some DUBs have high molecular weight, which leads to difficulties in crystal formation and obtaining complete crystal structure; 3) DUBs may undergo conformational changes after ubiquitin binding, suggesting the flexibility of their active sites, which poses challenges to the prediction and computer simulation of small molecules and leads to inefficiency; 4) The mechanism of DUBs regulation is complex, involving allosteric regulation of catalytic activity and/or substrate-mediated catalysis.

## Conclusions and perspectives

In this review, we have shown that DUBs are attractive drug targets in hematological malignancies, and some DUB inhibitors have shown favorable anti-hematologic effects both in vitro and in vivo. However, some DUBs, such as USP9X and USP22, display both oncogenic and tumor suppressor properties in hematological malignancies. To further clarify the role of DUBs in hematological malignancies, more studies should be focused on the location and substrate specificity of DUBs, as well as their interaction with other oncoproteins. Moreover, further analysis of the ubiquitin chain cleavage mode, the chain linkage specificity of DUBs, and the ubiquitin chain architecture on substrate proteins will help to understand the different biological functions of DUBs. In addition, understanding the role of DUBs in hematopoietic stem cells (HSCs) maintenance and differentiation would also contribute to elucidation of the pathogenesis of hematological malignancies.

DUBs are involved in a variety of cellular life activities, including DNA methylation, DNA damage repair, survival, differentiation, and apoptosis. This functional diversity indicates: 1) DUBs inhibition may overcome drug resistance and relapse, which are the major problems in the clinical treatment of hematological malignancies; 2) Targeted inhibition of some DUBs can eliminate leukemia stem cells and minimal residual disease; 3) Therapeutic DUB inhibitors can be used in combination with other anticancer therapies.

To improve the specificity of DUB inhibitors, allosteric inhibitors might be an attractive alternative, or by blocking the interactions between DUBs and specific substrate.

Overall, advances in the field of deubiquitinases further pave the way for targeted therapies, and we believe that the development of DUB inhibitors could bring clinical benefits to patients with hematological malignancies.

## Data Availability

Not applicable.

## Abbreviations

DUB: Deubiquitinase
UPS: Ubiquitin-proteasome system
MM: Multiple myeloma
MCL: Mantle-cell lymphoma
USP: Ubiquitin-specific protease
UCH: Ubiquitin carboxy-terminal hydrolase
OTU: Ovarian tumor-related protease
MJD: Machado-Joseph disease protein domain protease
JAMM: JAB1/PAB1/MPN-domain containing metallo-enzyme
MINDY: Motif interacting with Ub-containing novel DUB family
HSC: Hematopoietic stem cell
CML: Chronic myeloid leukemia
TKI: Tyrosine kinase inhibitor
TFR: Treatment-free remission
PTEN: Phosphatase and tensin homolog
SKP2: S-phase kinase-associated protein 2
AML: Acute myeloid leukemia
NPM: Nucleophosmin
HSPC: Hematopoietic stem and progenitor cell
RA: All-trans-retinoic acid
APL: Acute promyelocytic leukemia
CNS: Central nervous system
CAR: Chimeric antigen receptor
ALL: Acute lymphoblastic leukemia
CLL: Chronic lymphocytic leukemia
ATM: Ataxia telangiectasia mutated
OS: Overall survival
ASCT: Autologous stem cell transplantation
HL: Hodgkin lymphoma
cHL: Classical Hodgkin lymphoma
NHL: Non-hodgkin lymphoma
DLBCL: Diffuse large B cell lymphoma
FL: Follicular lymphoma
MLT: Malignant lymphomas of thymus
NKTL: Natural killer /T cell lymphoma
GCB: Germinal center B-cell-like
IPI: International prognostic index
BL: Burkitt’s lymphoma
ALCL: Anaplastic large-cell lymphoma
ATLL: Adult T-cell leukemia/lymphoma
